# Antithrombin use and 28-day in-hospital mortality among severe-burn patients: an observational nationwide study

**DOI:** 10.1186/s13613-017-0244-y

**Published:** 2017-02-20

**Authors:** Takashi Tagami, Hiroki Matsui, Yuuta Moroe, Reo Fukuda, Ami Shibata, Chie Tanaka, Kyoko Unemoto, Kiyohide Fushimi, Hideo Yasunaga

**Affiliations:** 1 Department of Clinical Epidemiology and Health Economics, School of Public Health, Graduate School of Medicine, University of Tokyo, 7-3-1 Hongo, Bunkyo-ku, Tokyo, 1138555 Japan; 2 Department of Emergency and Critical Care Medicine, Nippon Medical School Tama Nagayama Hospital, Tokyo, Japan; 3 Department of Health Informatics and Policy, Tokyo Medical and Dental University Graduate School of Medicine, Tokyo, Japan

**Keywords:** Anticoagulant, Burns, Disseminated intravascular coagulation, Sepsis, Surgery

## Abstract

**Background:**

Previous studies have suggested that antithrombin may be beneficial for treating coagulopathy in patients with severe burns. However, robust evidence for this idea is lacking. We examined the hypothesis that antithrombin may be effective in treating patients with severe burns.

**Methods:**

We performed propensity score-matched analyses of the nationwide administrative Japanese Diagnosis Procedure Combination inpatient database. We identified patients with severe burns (burn index ≥ 10) who were recorded in the database from 1 July 2010 to 31 March 2013. We compared patients who were administered antithrombin within 2 days of admission (antithrombin group) and those who were not administered antithrombin (control group). The main outcomes were 28-day mortality and ventilator-free days (VFDs).

**Results:**

Eligible patients (*n* = 3223) from 618 hospitals were categorized into either an antithrombin group (*n* = 152) or control group (*n* = 3071). Propensity score matching created a matched cohort of 103 pairs with and without antithrombin. Twenty-eight-day mortality was lower in the antithrombin group compared with the control group in propensity-matched analysis (control vs. antithrombin, 47.6 vs. 33.0%; difference, 14.6%; 95% confidence interval [CI] 1.2–28.0). Cox regression analysis showed a significant difference in 28-day in-hospital mortality between the control and antithrombin propensity-matched groups (hazard ratio 0.58; 95% CI 0.37–0.90). There were significantly more VFDs in the antithrombin compared with the control group in propensity score-matched analysis (control vs. antithrombin, 12.6 vs. 16.4 days; difference −3.7; 95% CI −7.2 to −0.12).

**Conclusions:**

This nationwide database study demonstrated that antithrombin use may improve 28-day survival and increase VFDs in patients with severe burns. Further prospective studies are required to confirm these results.

**Electronic supplementary material:**

The online version of this article (doi:10.1186/s13613-017-0244-y) contains supplementary material, which is available to authorized users.

## Background

Burns severe enough to require medical attention are observed in nearly 11 million people and represent the fourth most common type of injury globally [1]. Although most burns are not fatal, more than 300,000 people worldwide do die each year of burn injuries [1]. The occurrence of coagulopathy in severe burns has been demonstrated in several studies [2–4]. Patients with severe burns are at high risk of inflammation and activation of the coagulation system, and a significant proportion of patients present with coagulopathy unrelated to fluid administration [5]. The emergence of coagulopathy is an independent predictor of 28-day mortality in patients with severe burns [2, 3, 5]. However, there is limited evidence regarding effective treatments for coagulopathy in severe-burn patients, and clear treatment recommendations in these patients are lacking [4].

Several previous studies suggested that antithrombin administration may be effective for treating coagulopathy in severe burns; however, there is currently no robust evidence to support this idea [6–12]. Low antithrombin levels have been identified as an independent predictor of mortality and duration of hospital stay [13]. Furthermore, a recent study reported therapeutic effects of antithrombin substitution on myocardial dysfunction and inflammation, as well as systemic fluid accumulation, following burns and smoke-inhalation injury in a clinically relevant animal model [8]. Lavrentieva et al. [9] conducted a pilot study (*n* = 31) to evaluate the efficacy of antithrombin in acute-phase burn-injury patients and found that antithrombin reduced hypercoagulation and improved organ function. However, the effect of antithrombin on mortality in patients with severe burns remains unknown.

We hypothesized that antithrombin could be effective for the treatment of patients with severe burns. The current study aimed to evaluate this hypothesis using data from a large nationwide inpatient database in Japan.

## Methods

### Ethical statement

This study was approved by the Institutional Review Board of the University of Tokyo, which waived the requirement for informed patient consent because of the anonymous nature of the data.

### Data source and variables

We analysed data from the Japanese Diagnosis Procedure Combination (DPC) database, the details of which have been described previously [14]. Briefly, the DPC database includes administrative claims and discharge abstract data for all inpatients discharged from over 1000 participating hospitals. It covers approximately 92% (244/266) of all tertiary-care emergency hospitals in Japan and 90% (90/100) of institutions certified for training burn specialists by the Japanese Society for Burn Injuries [15]. The database includes the following information for each patient: age; sex; primary diagnosis; comorbidities on admission and post-admission complications coded according to the International Classification of Diseases, 10th revision codes (ICD-10) and written in Japanese; medical procedures, including types of surgery, coded with original Japanese codes; daily records of drug administration and devices used; length of stay; and discharge status. The dates of hospital admission, surgery, bedside procedures, drugs administered, and hospital discharge are recorded using a uniform data-submission format [14–18]. To optimize the accuracy of the recorded diagnoses, the responsible physicians are obliged to record the diagnoses with reference to medical charts. The diagnostic records are also linked to the payment system, and the attending physicians are required to report objective evidence for the diagnosis of the disease for reimbursement of treatment [14–18]. Patient follow-up thus begins on the day of admission and ends on the date of discharge, either to home, to another hospital, or as a result of death. We could not follow up patients after discharge from hospital because this information was not available in the database [14–18].

The database also provides important clinical scores, including burn index and Japan Coma Scale (JCS) scores. Although the percentage of total burn-surface area was not available in the database, the burn index was recorded. The burn index takes into consideration both the surface area and thickness of the burn area: burn index = full thickness of total burn-surface area + 1/2 partial thickness of total burn-surface area [15, 19]. Furthermore, a previous large study suggested that burn index was a better predictor of mortality in burn patients than percentage of total body surface area [20]. The JCS correlates well with the Glasgow Coma Scale, and consciousness scored at 100 points on the JCS is equivalent to a score of 6–9 on the Glasgow Coma Scale. We categorized the JCS scores into four groups: 0 (alert); 1–3 (delirium); 10–30 (somnolence); and 100–300 (coma) [15–17]. To quantify the extent of the comorbidities, the ICD-10 code for each comorbidity was converted to a score, and the sum was used to calculate the Charlson Comorbidity Index (CCI), as described previously [15, 21, 22]. Briefly, the CCI provides a method of predicting mortality by classifying or weighting comorbidities and has been widely used by health researchers to measure case mixes and disease burdens [21]. We categorized hospital types as academic or non-academic. We defined hospital volume as the number of eligible patients treated in the current study and categorized hospital volume into tertiles (low, medium, and high).

### Patient selection and endpoints

We identified patients with severe burns (burn index ≥ 10) [15] who were recorded in the database from 1 July 2010 to 31 March 2013. We compared patients who were administered antithrombin within 2 days of admission (antithrombin group) and those who were not administered antithrombin (control group). The exclusion criteria for this study were: out-of-hospital cardiac arrest, discharge within 2 days after admission (to avoid immortal time bias), and readmitted patients with burns (to avoid patients with planned operations). All the antithrombin used in the current study was plasma-derived antithrombin. Recombinant antithrombin was not available in Japan during the study period, though recombinant antithrombin use was allowed in a limited number of hospitals from September 2015.

The primary endpoint of the study was all-cause 28-day in-hospital mortality. Ventilator-free days (VFDs) were a secondary endpoint in the propensity score-matched groups [23]. VFDs were defined as the number of days the patient remained alive without mechanical ventilation assistance during the first 28 days after admission; patients who died before day 28 were assigned 0 days [23]. We also evaluated incidence of post-admission complications including intracranial haemorrhage, gastrointestinal bleeding, thrombotic disorders (pulmonary embolism and deep venous thrombosis), and renal failure (acute renal failure and/or requirement of renal replacement therapy).

### Statistical analysis

We performed one-to-one matching analysis between the antithrombin and control groups, based on estimated propensity scores for each patient [24, 25]. We assessed the propensity score by fitting a logistic regression model for antithrombin use as a function of the patients’ demographic and clinical characteristics, and hospital factors, including the following, which were previously reported to have the potential to affect mortality and the extent of haemostatic changes in patients with severe burns: age; sex; burn index; CCI, level of consciousness on admission; hospital type and hospital volume; inhalation injury with/without requirement for mechanical ventilation; use of catecholamines (dobutamine, norepinephrine, and/or dopamine); treatment with albumin, haptoglobin, and other blood products; use of drugs for disseminated intravascular coagulation (heparin, thrombomodulin, gabexate mesilate, nafamostat mesilate, or ulinastatin); and requirement for escharotomy and debridement [1, 4, 5, 13, 15, 17, 19, 26–31]. The C-statistic for evaluating the goodness-of-fit was calculated. One-to-one matched analysis using nearest-neighbour matching was performed based on the estimated propensity scores of the patients; a match was accepted when a patient in the antithrombin group had an estimated score within 0.2 standard deviations of a patient in the control group [24]. We examined the balance in baseline variables using standardized differences, where an absolute value <10% was regarded as balanced [24]. Data are expressed as number (%) or mean (standard deviation). Continuous variables were compared between groups using *t* tests, and categorical variables were compared using χ^2^ test or Fisher’s exact tests. A value of *P* less than 0.05 was considered statistically significant. Cox regression analysis was used to assess differences in 28-day in-hospital mortality between propensity score-matched patients with and without antithrombin treatment [24]. We performed logistic regression analysis fitted with generalized estimating equations to examine the association between antithrombin use and 28-day survival accounting for the paired nature of the propensity score-matched patients [24]. Because of its retrospective nature, we did not perform sample-size estimation for the current study. All statistical analyses were performed using IBM SPSS version 22 (IBM Corp., Armonk, NY, USA).

## Results

### Patients

**Fig. 1:**
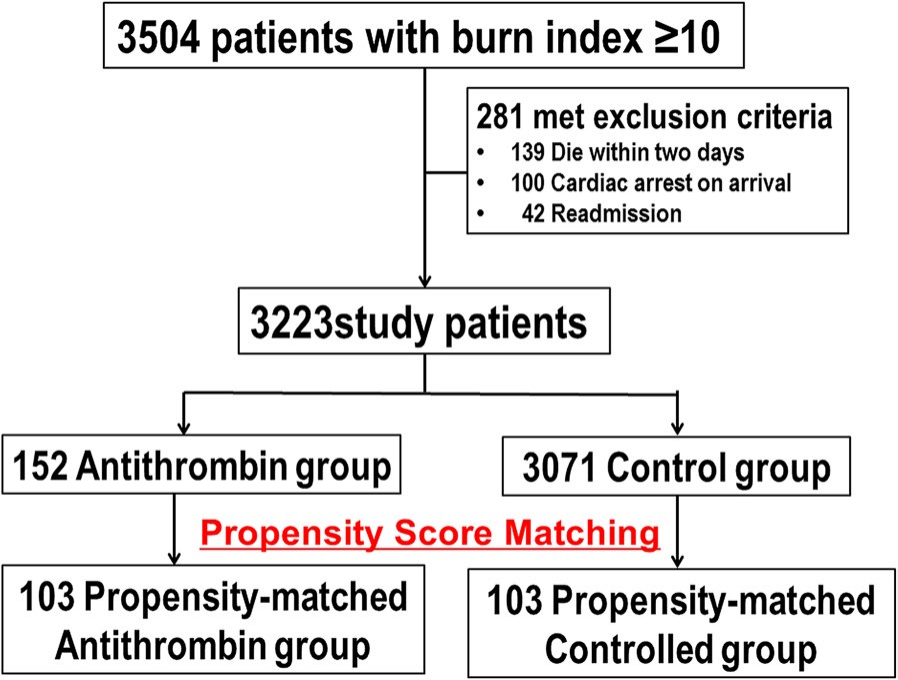
Patient selection

A total of 3223 patients treated at 618 hospitals during the 33-month study period were identified as eligible. Patients were divided into an antithrombin group (*n* = 152) and a control group (*n* = 3071), from which 103 propensity score-matched pairs were generated (Fig. 1). The C-statistic indicated a goodness-of-fit of 0.95 for the propensity score model.

Table 1 shows the baseline characteristics of the unmatched and propensity score-matched groups. Patients were more likely to receive antithrombin if they had severe burns, a higher burn index, and more requirements for mechanical ventilation, catecholamines, and other treatments, according to comparisons between unmatched groups. After propensity score matching, most of the baseline patient characteristics were well balanced between the groups. There was no significant difference in associated trauma lesions between the two groups (Additional file 1: Table S1). The median dose of antithrombin administered in the antithrombin group was 1500 U/day (minimum 500 U/day, maximum 3000 U/day; 90 percentiles 1500–3000 U/day) for 3 days (minimum 1 days, maximum 48 days; 90 percentiles 2–17 days). The median length of hospital stay among eligible patients was 54 days.

**Table 1:** Baseline patient characteristics in the unmatched and propensity score-matched groups

Variable	Unmatched groups			Matched groups		
	Control (*n* = 3071)	Antithrombin (*n* = 152)	Standardized differences (%)	Control (*n* = 103)	Antithrombin (*n* = 103)	Standardized differences (%)
Age, years (SD)	56.2 (26.5)	62.7 (22.0)	−27.0	63.6 (21.3)	61.6 (21.8)	9.2
Sex (male)	1887 (61.4)	97 (63.8)	−4.9	66 (64.1)	68 (66.0)	−4.1
Burn index, mean (SD)	21.7 (17.1)	40.5 (23.4)	−91.8	38.5 (23.9)	37.1 (22.1)	6.0
Inhalation injury without mechanical ventilation	188 (6.1)	1 (0.7)	30.5	1 (1.0)	1 (1.0)	0.0
Mechanical ventilation	651 (21.2)	124 (81.6)	−151.6	83 (80.6)	79 (76.7)	9.5
Charlson Comorbidity Index ≥ 1	409 (13.3)	11 (7.2)	20.1	8 (7.8)	7 (6.8)	3.7
Academic hospital	1097 (35.7)	108 (71.1)	−75.7	67 (65.0)	69 (67.0)	−4.1
Hospital volume, cases						
Low	1163 (37.9)	14 (9.2)	71.8	13 (12.6)	12 (11.7)	3.0
Medium	942 (30.7)	59 (38.8)	−17.2	44 (42.7)	38 (36.9)	11.9
High	966 (31.5)	79 (52.0)	−42.5	46 (44.7)	53 (51.5)	−13.6
Consciousness level						0.0
Alert	2230 (72.6)	62 (40.8)	67.8	40 (38.8)	45 (43.7)	−9.9
Delirium	459 (14.9)	38 (25.0)	−25.3	34 (33.0)	27 (26.2)	14.9
Somnolence	129 (4.2)	15 (9.9)	−22.3	9 (8.7)	11 (10.7)	−6.6
Coma	253 (8.2)	37 (24.3)	−44.7	20 (19.4)	20 (19.4)	0.0
Antibiotics use	1229 (40.0)	114 (75.0)	−75.7	76 (73.8)	74 (71.8)	4.4
Catecholamines						
Dopamine use	233 (7.6)	44 (28.9)	−57.5	31 (30.1)	27 (26.2)	8.6
Dobutamine use	47 (1.5)	13 (8.6)	−32.5	6 (5.8)	9 (8.7)	−11.2
Noradrenaline use	139 (4.5)	28 (18.4)	−44.7	15 (14.6)	14 (13.6)	2.8
Escharotomy performed	130 (4.2)	23 (15.1)	−37.5	16 (15.5)	18 (17.5)	−5.2
Debridement performed	127 (4.1)	10 (6.6)	−10.9	7 (6.8)	6 (5.8)	4.0
Red blood cell use	109 (3.5)	39 (25.7)	−65.9	23 (22.3)	21 (20.4)	4.7
Fresh-frozen plasma use	217 (7.1)	81 (53.3)	−116.5	44 (42.7)	45 (43.7)	−2.0
Platelet use	19 (0.6)	15 (9.9)	−42.4	5 (4.9)	4 (3.9)	4.8
Albumin use	608 (19.8)	133 (87.5)	−184.9	89 (86.4)	87 (84.5)	5.5
Haptoglobin use	196 (6.4)	67 (44.1)	−96.3	31 (30.1)	31 (30.1)	0.0
Heparin use	644 (21.0)	110 (72.4)	−120.2	73 (70.9)	72 (69.9)	2.1
Thrombomodulin use	14 (0.5)	30 (19.7)	−67.5	4 (3.9)	5 (4.9)	−4.8
Gabexate mesilate use	25 (0.8)	15 (9.9)	−41.1	5 (4.9)	6 (5.8)	−4.3
Nafamostat mesilate use	57 (1.9)	19 (12.5)	−42.1	16 (15.5)	16 (15.5)	0.0
Ulinastatin use	50 (1.6)	20 (13.2)	−45.2	9 (8.7)	11 (10.7)	−6.6

*SD* standard deviation

### Endpoints

**Fig. 2:**
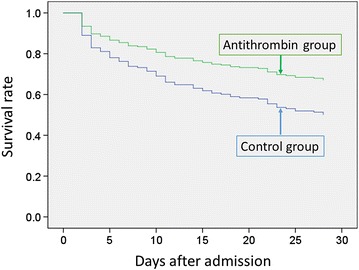
Survival plots for propensity-matched groups of patients treated with or without antithrombin. The survival rate was higher in the antithrombin group compared with the control group (hazard ratio 0.58; 95% CI 0.37–0.90)

Overall 28-day mortality was 14.7% (475/3223) in this cohort. Twenty-eight-day mortality was higher in the antithrombin group compared with the control group in unmatched analysis (control vs. antithrombin, 13.5 vs. 39.5%; difference −26.0%; 95% confidence interval [CI] −31.7 to −20.2), but 28-day mortality was lower in the antithrombin compared with the control group in propensity-matched analysis (control vs. antithrombin, 47.6 vs. 33.0%; difference 14.6%; 95% CI 1.2–28.0) (Table 2). Cox regression analysis showed a significant difference in 28-day in-hospital mortality between the control and antithrombin propensity-matched groups (hazard ratio 0.58; 95% CI 0.37–0.90) (Fig. 2). Logistic regression analyses using generalized estimating equations accounting for the paired nature of the propensity score-matched patients showed a significant association between antithrombin use and 28-day mortality in the propensity-matched groups (odds ratio 0.54; 95% CI 0.31–0.95).

**Table 2:** Comparisons of 28-day in-hospital mortality rates between the groups

	Control	Antithrombin	Difference (95% CI)	*P* value
Unmatched groups	13.5% (415/3071)	39.5% (60/152)	−26.0% (−31.7 to −20.2)	<0.001
Propensity-matched groups	47.6% (49/103)	33.0% (34/103)	14.7% (1.2 to 28.0)	0.03

There were significantly more VFDs in the antithrombin group compared with the propensity score-matched control group (control vs. antithrombin, 12.6 vs. 16.4 days; difference −3.7; 95% CI −7.2 to −0.12) (Table 3). There was no significant difference in the incidence of post-admission complications between patients with and without antithrombin in the matched groups.

**Table 3:** Secondary endpoints in unmatched and matched groups

Outcomes	Unmatched groups			Matched groups		
	Control (*n* = 3071)	Antithrombin (*n* = 152)	*P* value	Control (*n* = 103)	Antithrombin (*n* = 103)	*P* value
Ventilator-free days	23.1 (10.0)	15.2 (13.2)	<0.001	12.6 (12.9)	16.4 (12.8)	0.04
Post-admission complications						
Intracranial haemorrhage	9 (0.3)	2 (1.3)	0.09	0 (0)	0 (0)	NA
Gastrointestinal bleeding	19 (0.6)	0 (0)	1.0	2 (1.9)	0 (0)	0.50
Thrombotic disorders	22 (0.7)	0 (0)	0.62	1 (1.0)	0 (0)	1.0
Acute renal failure	116 (3.8)	26 (17.1)	<0.001	13 (12.6)	18 (17.6)	0.43

## Discussion

In the current study, we analysed data from a Japanese nationwide in-hospital database and found a significant association between antithrombin use and reduction in 28-day mortality in patients with severe burns. This finding was robust with regard to the results obtained by logistic regression and survival analyses. Antithrombin use was also associated with more VFDs in these patients.

The strengths of the current study included its use of a nationwide database and controlling for major factors (e.g. age, size and depth of the burn, and the existence of inhalation injury) that could potentially affect mortality and the extent of haemostatic changes in patients. The extent of haemostatic change is associated with the severity of the burn [26]; although most patients with mild burns have no systemic coagulation changes, patients with severe burns frequently develop coagulopathy [26]. We therefore only included patients with severe burns (i.e. burn index >10) [15] in the current study. In addition to burn size, full-thickness burns and inhalation trauma are also associated with the occurrence and severity of systemic coagulopathy [5, 32]. The baseline patient characteristics in the unmatched groups suggested that antithrombin use was higher in patients with severe burns (e.g. those with higher mortality and higher burn index and those requiring mechanical ventilation, catecholamines, and other treatments). However, we ensured the comparabilities of the two groups by propensity score matching, which provides a sound method for constructing a randomized experiment-like situation by comparing groups with similar observed characteristics, without specifying the relationships between confounders and outcomes [24]. We included factors that could potentially affect mortality and the extent of haemostatic changes in patients with severe burns when estimating propensity scores, such as age, burn-surface area and depth (i.e. burn index), comorbidities, and mechanical ventilation status [15, 19, 28]. After one-to-one propensity score matching, the two groups (i.e. antithrombin vs. control) were well balanced in most of the measured variables. Our results suggested that patients with severe burns who received antithrombin were less likely to die and had more VFDs than those who did not receive antithrombin. There was no significant difference in the incidence of post-admission complications between two groups. The current results were compatible with those of previous sepsis studies, which found that antithrombin did not increase bleeding risk, at least at the dose used in Japan (i.e. 1500 U/day) [33, 34].

Several previous studies suggested that severe thermal injury is associated with early activation of the coagulation cascade and the emergence of coagulopathy, which worsens the outcome of burns patients [2–5]. Interestingly, several similar patterns of procoagulant and antifibrinolytic changes, as well as natural anticoagulant system impairments, were witnessed in both early-stage severe-burn patients and sepsis patients [2, 3, 9, 35]. The guidelines of the Japanese Society of Thrombosis Haemostasis [36] and the Japanese Society of Intensive Care Medicine [37] both recommend the use of supplemental antithrombin in patients with sepsis-associated coagulopathy, and anticoagulant therapy, including antithrombin, is thus commonly used in clinical practice in Japan [34]. Although no large randomized trials have suggested the effectiveness of antithrombin use, recent nationwide database studies suggested an association between lower in-hospital mortality and supplemental antithrombin administration among patients with pneumonia and abdominal sepsis [16, 29].

Several treatment guidelines for burns, including Japanese guidelines [38], do not address or recommend the use of anticoagulation treatments for severe-burn patients, because of a lack of scientific evidence [39]. European guidelines for bleeding care in trauma management suggest that antithrombin is not recommended in acutely bleeding trauma patients because of the increased risk of bleeding events and its failure to reduce overall mortality [40]. Although numerous studies have explored coagulopathy in sepsis patients, few have evaluated coagulopathy in severe-burn patients in any detail [4]. Moreover, clear definitions or diagnostic criteria for coagulopathy after burns are currently absent. A lack of simple and easy-to-interpret diagnostic tests may thus disguise the true incidence of coagulopathy in patients with severe burns [4]. Antithrombin deficiency is common after burns and is related to total burn-surface area and inhalation injury, increased mortality, and longer hospital stay [13]. However, treatments for coagulopathy after severe burns may be less popular than treatments for sepsis-associated coagulopathy. Indeed, the current nationwide study, using data from a country where antithrombin is commonly used as anticoagulant therapy in clinical practice [34], found that antithrombin was only administered to 152 of 3223 eligible severe-burn patients. The current results may thus support further prospective studies in this area.

A previous study suggested that early-onset coagulopathy was associated with prolonged mechanical ventilation [32], while the current results indicated that antithrombin use was associated with more VFDs. This may be because antithrombin acts as an anticoagulant with anti-inflammatory properties. A previous observational study [41] showed that pulmonary coagulopathy seemed intrinsic to burn injuries and inhalation trauma, and concluded that patients with burn injuries and inhalation trauma requiring mechanical ventilation showed a distinct and sustained procoagulant and antifibrinolytic shift in the pulmonary compartment, indicating that pulmonary coagulopathy could be an important therapeutic target in these patients. Several recent experimental studies suggested that antithrombin attenuates myocardial dysfunction and pulmonary vascular leakage in patients with severe burns and inhalation injury [8, 42]. Rehberg et al. [42] showed an interaction between antithrombin and neutrophils in vivo and demonstrated its pathophysiological role in vascular leakage and the therapeutic potential of antithrombin in a sheep model. Rehberg et al. [8] also demonstrated therapeutic effects of antithrombin in terms of myocardial dysfunction and inflammation, as well as systemic fluid accumulation following burns and smoke-inhalation injury in a sheep model. Based on the current and previous studies [8, 41, 42], supplemental antithrombin treatment may represent a valuable therapeutic approach for cardiovascular dysfunction and inflammation after burn and smoke-inhalation injury. However, further studies are required to confirm these results.

This study had some limitations. First, the current study was retrospective and observational in nature, without randomization. Even though propensity score matching was used to adjust for differences in baseline characteristics and disease severity, including factors that could potentially affect mortality and the extent of haemostatic change in patients with severe burns, there may still have been bias in the form of unmeasured confounders. Possible confounding parameters include laboratory-based coagulation tests, which indicate the level of coagulopathy (e.g. international normalized ratio, activated partial thromboplastin time, antithrombin), systemic inflammatory syndrome response, disseminated intravascular coagulation scoring system, blood gas analysis, serum lactate level, fluid balance per day, haemodilution caused by large amounts of resuscitation fluids together with hypothermia, and type of hospital admission (e.g. general burn ward or advanced burn unit). Unfortunately, these data were not available from the DPC database. However, previous studies suggested that the severity of coagulopathy was correlated with the extent and depth of the burn and the existence of inhalation injury [4, 26], and we were able to match these factors using propensity scoring, when comparing the outcomes between the antithrombin and control groups. Second, this study only evaluated the association between early-phase antithrombin use and outcomes and did not evaluate the effect of later-phase antithrombin use (e.g. antithrombin use after sepsis-associated disseminated intravascular coagulation due to burn-wound infection). Third, we could not identify the cause of death or long-term outcome from the current DPC database and therefore could not speculate on the factors responsible for the improved 28-day mortality in the antithrombin group compared with the control group. Fourth, we did not determine whether the replacement of coagulation factors using fresh-frozen plasma was superior to antithrombin provision, though this research question should be evaluated in future studies.

## Conclusions

 Analysis of this nationwide database demonstrated that antithrombin use may improve 28-day survival in severe-burn patients. However, further studies are required to confirm these results.

## Additional file

**Additional file 1: Table S1.** Frequencies of coexisting traumas.

## Abbreviations

CCI: Charlson Comorbidity Index
DPC: Diagnosis Procedure Combination
ICD: International Classification of Diseases
JCS: Japan Coma Scale
VFDs: ventilator-free days
