# Supplementary material for: Antithrombin use and 28-day in-hospital mortality among severe-burn patients: an observational nationwide study
Source: Ann Intensive Care. 2017 Feb 20;7:18. doi: 10.1186/s13613-017-0244-y (PMC5318343; doi:10.1186/s13613-017-0244-y)
Supplement: Supplementary file 1 — Additional file 1: Table S1. Frequencies of coexisting traumas. [file 13613_2017_244_MOESM1_ESM.docx]

**Additional file 1: Table S1.** Frequencies of coexisting traumas

|  | **Control**  **(n = 3071)** | | **Antithrombin**  **(n = 152)** | | **p value** |
| --- | --- | --- | --- | --- | --- |
| **Head trauma** | 12 | (0.4) | 0 | (0.0) | 1.00 |
| **Chest trauma** | 5 | (0.2) | 1 | (0.7) | 0.25 |
| **Abdominal trauma** | 3 | (0.1) | 0 | (0.0) | 1.00 |
| **Pelvic fracture** | 6 | (0.2) | 1 | (0.7) | 0.29 |
| **Spinal fracture** | 7 | (0.2) | 1 | (0.7) | 0.32 |

Data are presented as n (%).
